# Derivation of Induced Pluripotent Stem Cells from Human Peripheral Blood T Lymphocytes

**DOI:** 10.1371/journal.pone.0011373

**Published:** 2010-06-29

**Authors:** Matthew E. Brown, Elizabeth Rondon, Deepika Rajesh, Amanda Mack, Rachel Lewis, Xuezhu Feng, Laura Jo Zitur, Randall D. Learish, Emile F. Nuwaysir

**Affiliations:** Department of Research and Development, Cellular Dynamics International, Inc., Madison, Wisconsin, United States of America; New York University, United States of America

## Abstract

Induced pluripotent stem cells (iPSCs) hold enormous potential for the development of personalized *in vitro* disease models, genomic health analyses, and autologous cell therapy. Here we describe the generation of T lymphocyte-derived iPSCs from small, clinically advantageous volumes of non-mobilized peripheral blood. These T-cell derived iPSCs (“TiPS”) retain a normal karyotype and genetic identity to the donor. They share common characteristics with human embryonic stem cells (hESCs) with respect to morphology, pluripotency-associated marker expression and capacity to generate neurons, cardiomyocytes, and hematopoietic progenitor cells. Additionally, they retain their characteristic T-cell receptor (TCR) gene rearrangements, a property which could be exploited for iPSC clone tracking and T-cell development studies. Reprogramming T-cells procured in a minimally invasive manner can be used to characterize and expand donor specific iPSCs, and control their differentiation into specific lineages.

## Introduction

*In vitro* reprogramming of somatic cells to an undifferentiated pluripotent state by viral transfer of defined factors such as *SOX2, OCT4, NANOG* and *LIN28* or *SOX2, OCT4, c-Myc*, and *KLF4* [1], [2] has opened the way for the generation of patient-specific human iPSCs using multiple cell types [3], [4]. This premise has been further advanced by derivation of iPSCs via transient expression of genes or by using protein transduction of appropriate transcription factors [5], [6]. To date, the majority of iPSC research in humans has focused on fibroblasts as a cell source. While fibroblasts offer certain advantages as a starting material due to their commercial availability and ease of gene delivery, they are suboptimal for large-scale clinical derivation of iPSC lines due to the need for invasive skin biopsies and the difficulty of establishing stable lines from primary tissue. Non-mobilized peripheral blood is perhaps the ideal cell source for reprogramming due to the ease of obtaining patient samples [7]. Additionally, large numbers of frozen blood samples, from living and deceased donors, are stored in biorepositories worldwide [8].

Investigators have recently reported successful reprogramming of primary CD34^+^ hematopoietic progenitor cells from both mobilized and non-mobilized blood donors [3], [9]. These findings represent an important advance in iPSC research, however, non-mobilized adult peripheral blood contains approximately 100–1000 CD34^+^ cells/ml [10], [11] making these rare progenitors a challenging cell source for iPSC line derivation from small blood volumes. As an alternative, more abundant and tractable blood cell source we report the derivation of iPSCs from T lymphocytes obtained from the equivalent of 1 ml whole blood. These T-cell derived iPSCs (“TiPS”) share essential characteristics with hESCs as well as fibroblast-derived iPSC lines. Additionally, they retain their characteristic T-cell receptor (TCR) gene rearrangements, a property which could be exploited, for example, as a genetic tracking marker or in re-differentiation experiments to study human T-cell development.

## Results and Discussion

**Figure 1:**
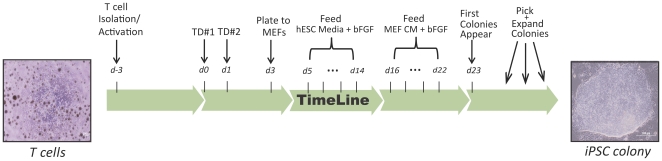
Overview of T-cell reprogramming process, beginning with activated T-cells and resulting in iPSC colonies with hESC-like morphology. T-cell and iPSC colony images were acquired on an Olympus IX71 microscope with 10× and 20× objectives, respectively.

**Figure 2:**
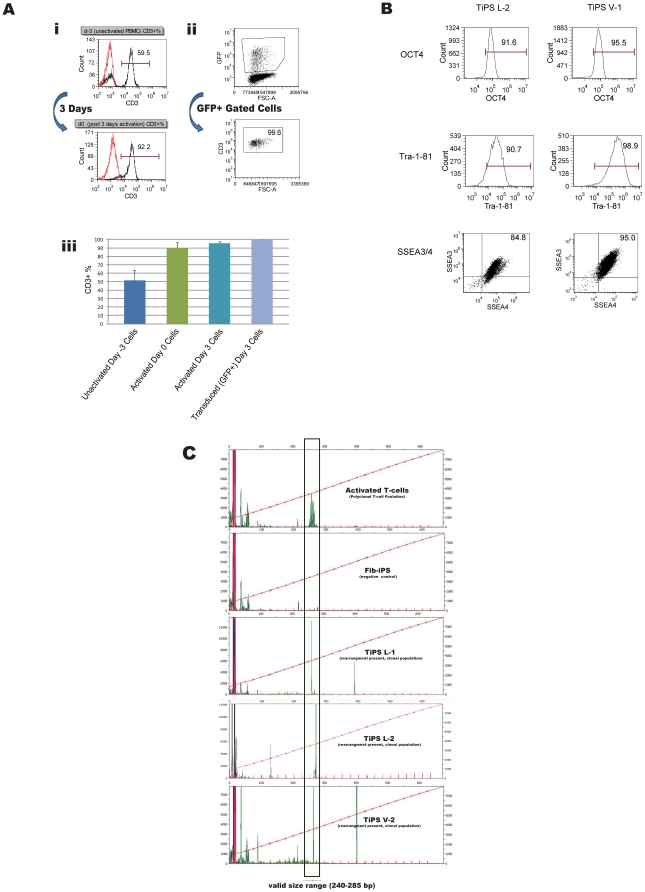
Derivation and characterization of induced pluripotent stem cells from human T-cells. (A) Flow cytometric analysis of input cell source CD3 surface expression. (i) CD3 surface expression on day -3 non-activated PBMCs and day 0 activated T-cells from the PBMC population in a representative donor. (ii) CD3 expression gated on the transduced (GFP^+^) cell population 72 hours post-transduction in a representative donor to demonstrate preferential transduction of CD3^+^ cells. (iii) Histogram representation of the above metrics (i-ii) in an average of 10 donor Vacutainer-derived samples. (B) Flow cytometric analysis of hESC pluripotency markers OCT4, Tra-1-81, SSEA-3 and SSEA-4 in representative leukapheresis (“TiPS L-2”) and Vacutainer (“TiPS V-1”) derived TiPS lines. (C) T-cell receptor (TCR) β chain rearrangement analysis using multiplexed PCR primers targeted to conserved regions within the V-J region of the TCR β locus. Polyclonal starting T-cell populations are represented by a bell-shaped curve of amplicon peaks within the valid fragment size range on the electropherogram. Fibroblast (non-T-cell) iPS cells (“Fib-iPS”) lack germline rearrangement at the TCR β locus and serve as a negative control. The clonally derived TiPS lines (representative data from two leukapheresis lines and one Vacutainer line, “TiPS L-1”, “TiPS L-2” and “TiPS V-2”, respectively) show one distinct peak of defined size. DNA fragment analysis was performed on an ABI 3730 DNA analyzer.

T-cells are well suited as a starting material for reprogramming due to their abundance in whole blood (∼6.5×10^5^–3.1×10^6^/ml in healthy adults) [12] and ease of culture using well-established protocols [13], [14]. To facilitate T-cell proliferation and efficient retroviral transduction, peripheral blood mononuclear cells (PBMCs) were isolated from a leukapheresis or a standard venipuncture (Vacutainer© CPT tube) and cultured in serum-free media with IL-2 and anti-CD3 antibody (Figure 1). This led to preferential expansion of mature CD3+ T-cells consisting of an average day 3 CD3^+^ purity of 90% +/− 7% (Figure 2A).

Activated T-cell enriched populations containing 1×10^6^ cells were subjected to two rounds (at day 0 and 1) of retroviral transduction with four separate vectors, each encoding one of the reprogramming factors (*SOX2*, *OCT4*, *c-Myc*, or *KLF4*) linked to a fluorescent marker gene. Transduction efficiency was assessed at day 3 by fluorescence microscopy and flow cytometry. Staining for CD3 showed the transduced population to be 99% +/− 1% CD3^+^ (Figure 2A).

The population of cells containing the transduced T-cells was placed on irradiated mouse embryonic fibroblasts (MEFs) in hESC medium supplemented with 100 ng/ml basic fibroblast growth factor (bFGF). iPSC colonies were observed beginning at day 23. Reprogramming efficiencies of T-cells were estimated by dividing the number of colonies with hESC-like morphology by the input number of transduced cells and determined to be approximately 0.01%, similar to published fibroblast and CD34^+^ cell efficiencies [1], [3].

TiPS were generated from both leukapheresis samples (from a male Hispanic adult, lines denoted “TiPS-L”) and whole blood Vacutainer samples (from a male Caucasian adult, lines denoted “TiPS-V”). In each case, reprogramming was achieved using an input cell number equivalent to the amount of T-cells in 1 ml whole blood. Colonies displaying hESC morphology were expanded on MEFs and the clones were successfully maintained under feeder-free conditions using mTeSR media and Matrigel coated plates.

We performed DNA fingerprinting to verify that TiPS shared a genetic background with the starting donor T-cell population and to rule out cell line cross-contamination (Figure S1). Pluripotency was verified by expression of hESC pluripotency markers SSEA-3, SSEA-4, Tra-1-81, and OCT4 using flow cytometry (Figure 2B) and alkaline phosphatase staining (Figure S2). We confirmed the TiPS lines' T-cell origin via multiplex PCR detection of TCR β chain rearrangements (Figure 2C).

**Figure 3:**
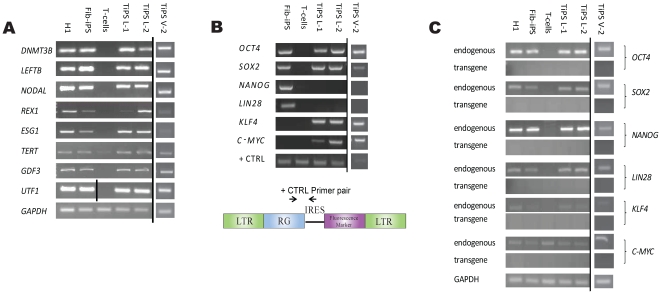
Characterization of induced pluripotent stem cells from human T-cells. (A) RT-PCR analysis of representative leukapheresis (“TiPS L-1” and “TiPS L-2”) and Vacutainer (“TiPS V-2”) derived TiPS cell lines for expression of hES cell-marker genes *DNMT38*, *LEFTB*, *NODAL*, *REX1*, *ESG1*, *TERT*, *GDF3*, and *UTF1*. GAPDH was used as positive loading control for each sample. (B) PCR analysis of genomic DNA confirms integration of the transgenes. Forward primers for the reprogramming gene (“RG”) of interest and reverse primers for the IRES were utilized. *OCT4* forward and reverse primers were used as the PCR reaction positive control, as shown in vector map. (C) RT-PCR analysis of TiPS cell lines shows silencing of the exogenous transgenes, with GAPDH as positive control for each sample. In (A) and (C) hESC line H1 and in (A-C) a fibroblast derived iPSC line (“Fib-iPS”) served as positive cell controls, and activated donor T-cells served as a negative cell control. In (A-C) images were modified with Photoshop software to combine gels and remove redundant control data from separate experiments.

RT-PCR was performed to confirm expression of hESC genetic markers *DNMT38*, *LEFT8*, *NODAL*, *REX1*, *ESG1*, *TERT*, *GDF3*, and *UTF1* (Figure 3A). Further characterization demonstrated integration of the transgenes into the host genome as well as their silencing following successful reprogramming (Figures 3B, C). TiPS were similar to both the hESC line H1 and to fibroblast-derived iPSC line controls in all of the above assays. Additionally, lines were karyotypically normal after multiple passages and have been propagated for over 30 passages in culture while retaining a normal karyotype (Figure S3).

**Figure 4:**
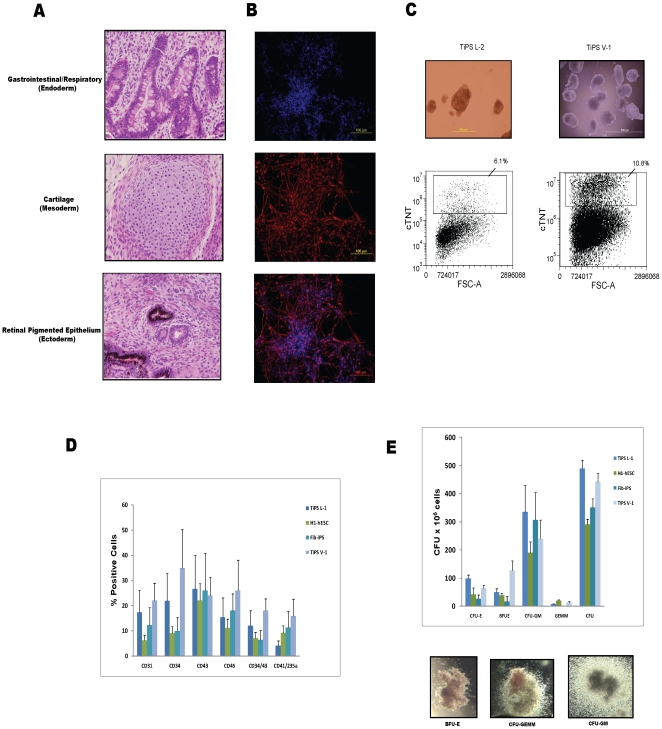
*In vivo* and *in vitro* differentiation potential of TiPS cell lines. (A) Teratoma formation shows *in vivo* differentiation potential. TiPS cells injected into SCID/beige mice formed teratomas at 5 to 12 weeks. Hematoxylin and eosin staining shows tissues consistent with derivation from the three primary germ layers including simple epithelium with goblet cells indicating gastrointestinal or respiratory tissue (endoderm), cartilage (mesoderm) and retinal pigmented epithelium (ectoderm). Representative images from TiPS L-2 cell line were acquired using an Olympus IX71 microscope using a 40× objective. (B) *In vitro* differentiation into neurons. TiPS L-2 cells were induced into neuronal differentiation as aggregates then stained for neuronal marker beta III-tubulin with an Alexa Fluor 594 secondary antibody; cell nuclei were counterstained with Hoechst stain. Images were acquired using a 20× objective. Contrast was adjusted and images were merged using Image J software. (C) Cardiac induction of TiPS cells via cell aggregate method. Cell aggregates contain beating cardiac troponin T (cTNT)-positive cardiomyocytes at days 14 to 15. Flow data from representative samples is shown. Images were acquired using a 10× objective. (D) In vitro differentiation into hematopoietic progenitor cells. Hematopoietic progenitor cells (HPCs) generated via a serum-free embryoid body (EB) differentiation protocol for 12 days in two TiPS lines compared to an hESC line (“H1”) and a fibroblast derived iPSC line (“Fib-iPS”). HPCs were quantified via flow cytometry by dissociating the EBs into single cells and staining with fluorochrome-conjugated monoclonal antibodies to CD34, CD45, CD43, CD31, CD41 and CD235a. (E) Hematopoietic clonogenic (CFU) assays were performed by placing EB differentiated and individualized cells into serum-free MethoCult media containing cytokines (SCF, G-CSF, GM-CSF, IL-3, IL-6, and EPO). Colonies were scored after 14 days of incubation according to morphologic criteria as erythroid (CFU-E/BFU-E), myeloid comprising macrophage (CFU-M), granulocyte (CFU-G), and granulocyte-macrophage (CFU-GM), and multilineage comprising granulocyte-erythroid-macrophage-megakaryocyte (CFU-GEMM) colonies. Total CFUs were quantified and representative images were acquired using an Olympus CKX41 microscope with a 2× objective.

Finally, the TiPS cell lines were evaluated to determine their *in vivo* and *in vitro* differentiation potential. All TiPS clones formed teratomas. The teratomas contained tissue consistent with derivation from all three primary germ layers (Figures 4A). The cell lines were also assessed for their capability to differentiate *in vitro* into ectodermal and mesodermal lineages in various directed differentiation protocols. The clones were able to generate neurons, beating cardiac troponin T-positive cardiomyocytes and multipotent granulocyte-erythroid-macrophage-megakaryocyte (GEMM) hematopoietic cells (Figures 4B, C, D, E).

**Figure 5:**
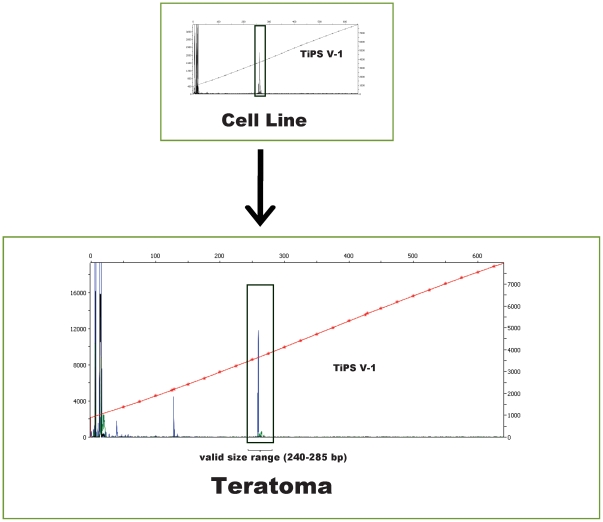
iPSC Clone Tracking. Genomic DNA was isolated from teratoma samples and compared with their parent cell lines for TCR β chain rearrangements. Representative data is shown from cell line TiPS V-1. The derivative teratoma harbors the clonal rearrangement of the parent cell line. PCR analysis was conducted using multiplexed primers targeted to conserved regions within the V-J region of the TCR β locus. DNA fragment analysis was performed on an ABI 3730 DNA analyzer. Background ≤1000 RFU.

A potential concern of T-cell derived iPSCs is the persistence of TCR gene rearrangements in the iPSC genome and their potential effect on subsequent differentiation. Though we did not observe any significant differences in differentiation potential between TiPS clones and hESC lines or fibroblast-derived iPSC lines (Figure 4D,E, additional data not shown) the effects of these genomic rearrangements on lymphoid differentiation remain to be investigated. TCR rearrangements may in fact prove advantageous in certain contexts, such as for iPSC clone tracking, as demonstrated by the detection of parent line clonal TCR β chain rearrangements in derivative teratomas (Figure 5). Further, upon *re*-differentiation into T-cells TiPS cells may bypass key steps in the canonical thymic development sequence due to the mechanism of TCR allelic exclusion caused by the expression of their pre-rearranged TCR genes. This phenomenon could be explored in T-cell development studies.

It should be noted that insertional mutagenesis and other potential disruptions of cellular function are possible when using a retroviral reprogramming protocol [15]. Recent advances in using episomal reprogramming methods may address these issues and efforts are in progress to reprogram T-cells via these alternative methods [5], [6]. Further, an interesting example of a potential therapeutic use for such episomally reprogrammed TiPS cells is as a source to differentiate integration-free hematopoietic stem cells bearing endogenous TCR genes specific for tumor-associated antigens [16].

Previous reports of reprogramming terminally differentiated B lymphocytes in mice required the addition, or knock-down, of cellular identity-associated transcription factors and used a doxycycline-inducible expression system [17]. Recently, a description of reprogramming murine T-cells was published necessitating a *p53* gene knock-out for successful iPSC generation [18]. Experiments involving manipulation of anti-proliferative pathways [19], [20], [21], [22] offer insights into the mechanisms of reprogramming and may significantly augment reprogramming efficiencies. However, none of the above mentioned manipulations appear to be a requirement for successful viral reprogramming of human T-cells. Additionally, our data, coupled with methodologies used in reprogramming adult CD34+ hematopoietic progenitor cells [3], [9], now afford a primary, human system to examine recent observations in the mouse system correlating differentiation stage of input cells with reprogramming efficiency [23].

We describe the derivation of iPSCs from small, clinically advantageous volumes of non-mobilized human peripheral blood. T-cells represent an abundant cell source for reprogramming which can be harvested from large numbers of donors in a minimally invasive manner and cultured via well-established protocols. In the experiments we have detailed, TiPS have similar characteristics and differentiation potential as hESC lines and fibroblast-derived iPSC lines. Additionally, TiPS provide a novel model with which to explore iPSC clone tracking, T-cell development and therapeutic applications of iPSC technology.

## Materials and Methods

### Cell Growth Media and Basic Fibroblast Growth Factor

iPSC lines were maintained using previously described methods [1]. Zebrafish bFGF was substituted for human bFGF in all experiments, as previously described [24].

### Fibroblast iPSC Lines

Control fibroblast-derived iPSC lines, referred to as “Fib-iPS”, were produced as previously described using IMR90 cells obtained from ATCC (Manassas, VA) [1].

### T-cell Activation and Expansion

Peripheral Blood Mononuclear Cells (PBMCs) were obtained from an HLA-A2 positive adult male Hispanic donor (“Donor L”) leukocyte pack (Biological Specialty Corp, Colmar, PA) processed with Lymphocyte Separation Medium (Cellgro, Manassas, VA). Additionally, whole blood samples were collected from a male Caucasian donor of unknown serotype (“Donor V”) via standard venipuncture in a Vacutainer© CPT™ tube (BD Biosciences, San Jose, CA) and PBMCs were collected by centrifugation according to the manufacturer's recommendations. Blood samples were obtained with written informed consent in accordance with the Declaration of Helsinki and Institutional Review Board approval from the Biological Specialty Corporation (Colmar, PA, USA). T-cells were expanded in freshly prepared AIM-V Medium (Invitrogen, Carlsbad, CA) supplemented with pen/strep/glutamine (Invitrogen) plus 300 IU/ml rhIL2 (Peprotech, Rocky Hill, NJ) and 10 ng/ml soluble anti-CD3 antibody (eBioscience, OKT3 clone, San Diego, CA) [25], [26] Proliferation was verified by CEDEX (Roche Innovatis, Bielefeld, Germany) cell count after 3 days in culture at which point cells were assayed for T-cell phenotype and then transduced with reprogramming factors.

### Transient Transfection for Retrovirus Production

Retrovirus was generated by transfecting 293T cells in a 10 cm plate at 70-80% confluence with 10 ug of retroviral vector (Moloney Murine Leukemia Virus) backbone encoding each of 4 reprogramming genes and a fluorescent marker gene (GFP or RFP), 3 ug of Gag-Pol, 1 ug of plasmid encoding a derivative of NFkB, and 1 ug of Vesicular Stomatitis Virus G protein using polyethylene imine (“PEI”) lipophilic reagent (40 ug/10 cm plate). After four hours, the medium was exchanged with 5 ml of DMEM (Invitrogen) plus 10% FBS (Hyclone, Waltham, MA) and 50 mM HEPES (Invitrogen). Viral supernatant was collected 48 hours post-transfection and passed through a 0.8 um pore size filter.

### Retroviral Transductions via Spinfection

One million activated donor cells per well were “spinfected” via centrifugation for 1.5 h×1000 g at 32°C in a mixture of the four retroviral supernatants plus 4 ug/ml polybrene (Sigma-Aldrich, St. Louis, MO), and 300 IU/ml rhIL-2. After spinfection the plates received a half-media exchange, and were incubated overnight. The next day the cells were harvested by centrifugation and spinfected a second time.

### Verification of T-cell Expansion and Transduction Efficiency

T-cell identity was verified 3 days after activation by flow cytometry surface staining with anti-CD3 antibodies (BD, clone HIT3a), as well as post-transduction to verify which cell population was transduced successfully. Samples were run on an Accuri (Ann Arbor, MI) flow cytometer. CEDEX cell counts were conducted on days 0, 3 and 4 to confirm expansion and thus amenability to MMLV retroviral infection (data not shown).

### Plating Transduced T-cells on MEFs

Seventy two hours post initial transduction, transduction success and efficiency estimates were verified by fluorescent microscopy and flow cytometry as listed above. 5×10^5^ transduced cells were added to 10 cm plate seeded with MEFs 1 to 3 days prior in a 50/50 media combination D10F:hESC without zbFGF (or additional cytokines). Cells were incubated and fed hESC media + 100 ng/ml zbFGF (first week) or MEF-conditioned media + 100 ng/ml zbFGF (thereafter) by half media exchange every other day. To avoid cell loss during feedings the plates were angled slightly for 10 minutes to allow the cells to settle and media was removed slowly from the media horizon.

### iPSC Colony Identification and Picking

Colonies with well-defined borders and typical hESC morphology began to appear around day 23. GFP and RFP silencing was verified by fluorescent microscopy and the number of colonies was counted to estimate reprogramming efficiency given the number of input plated cells. Colonies were manually harvested, transferred to MEFs, and expanded according to established protocols [7], [27] Estimates of reprogramming efficiency were obtained by dividing total number of putative iPSC colonies by the input number of transduced cells. Counts were ceased after colony harvest (day 25–30) to avoid the inclusion of false positive re-seeded colonies left behind from the harvest.

### DNA Fingerprinting

TiPS cell lines and donor PBMCs were sent to the University of Wisconsin Histocompatibility/Molecular Diagnostics Laboratory (Madison, WI) for short tandem repeat (STR) analysis. Genotypes for 8 STR loci were determined from TiPS cell sample DNA.

### Karyotyping

G-banding analysis was conducted by WiCell Research Institute (Madison, WI).

### T-cell Receptor β Chain Rearrangement Analysis

Genomic DNA was isolated per manufacturer's protocol (using the Qiagen DNeasy Blood and Tissue kit) from donor T-cells, the TiPS cell lines, and a fibroblast (non-T-cell) derived iPSC line used as a negative control. Additionally, DNA was isolated from frozen teratoma samples and parent cell lines by first dissolving tissue and cell samples in a buffer containing Tris, NaCl, EDTA, SDS and Proteinase K (Invitrogen). DNA was then precipitated with saturated NaCl and ethanol, and resuspended in water for PCR analysis. PCR was performed using a multiplex primer kit (Invivoscribe Technologies, San Diego, CA) specific for a majority of clonal TCR β chain rearrangements [28]. Capillary electrophoresis and PCR product fragment analysis was performed at the University of Wisconsin Biotechnology Center DNA Sequencing Core Facility (Madison, WI) using an ABI 3730 DNA analyzer. Data was analyzed using Peak Scanner software (ABI, Foster City, CA).

### Alkaline Phosphatase (AP) Staining

Confluent cells grown on MEFs were AP stained with Vector Blue Alkaline Phosphatase Substrate Kit III (Vector Laboratories, SK-5300, Burlingame, CA) according to the manufacturer's protocol.

### RT-PCR for Transgene and hESC Marker Gene Expression

Total RNA was isolated using the RNeasy Mini Kit (Qiagen, Germantown, MD) according to the manufacturer's protocol. First strand cDNA synthesis was carried out with oligo-dT primers (as described previously [1], [2]) using SuperScript III First Strand Synthesis kit (Invitrogen) according to the product protocol. cDNA was diluted 1:2 and PCR reactions were performed with GoTaq Green Master Mix (Promega, Madison, WI) using a Mastercyler (Eppendorf, Hauppauge, NY).

### PCR Analysis of Viral Integration

Genomic DNA was isolated from 1–5×10^6^ iPSCs using DNeasy Blood and Tissue kit (Qiagen) according to the manufacturer's protocol for cultured cells. Genomic DNA (5 ul) was used for PCR reactions to check for viral integration using GoTaq Green Master Mix (Promega). Specific primer sets were used that detect only the transgene and not the endogenous gene. Primers for endogenous *OCT4* served as a positive control for the reaction. Reactions were performed with primers as described previously [1], [2].

### Flow Cytometry: iPSC Line Intracellular and Surface Pluripotency Marker Characterization

TiPS maintained on Matrigel were harvested and stained for the presence of Tra-1-81(BD Pharmingen or Stemgent, San Diego, CA, both clone Tra-1-81), SSEA-3 (BD Pharmingen, clone MC631) and SSEA-4 (BD Pharmingen, clone MC813-70). Intracellular OCT4 (BD, clone 40/Oct-3) staining was performed on cells fixed with 2% paraformaldehyde and permeablized with PBS + 0.1% saponin. Cells were stained overnight and analyzed the next day on an Accuri flow cytometer.

### Hematopoietic Differentiation and Colony-Forming Unit Assays

Undifferentiated TiPS were adapted to feeder-free conditions on Matrigel coated plates and maintained using mTeSR medium (Stem Cell Technologies, Vancouver BC, Canada). The colonies were harvested using TrypLE (Invitrogen) and placed in serum-free embryoid body (EB) basal media [containing IMDM, NEAA, Glutamine (Invitrogen) and 20% BIT-9500 (Stem Cell Technologies) and ROCK inhibitor H1152] in low-attachment plates to facilitate aggregate formation. Following aggregate formation, the cells were placed in EB basal media supplemented with growth factors and cytokines: rhBMP-4 (R&D Systems, Minneapolis, MN), rhVEGF, zbFGF, rhFlt-3 ligand, rhIL-3, and rhGM-CSF (Invitrogen) for 12 days. The cells were harvested and the phenotype generated by each iPSC clone was assessed by surface staining for CD31, CD34, CD43, CD45, CD41 and CD235a by flow cytometry. The individualized cells were placed in MethoCult (Stem Cell Technologies) media for quantifying colony-forming units per the manufacturer's instructions.

### Assay for Teratoma Formation

Characterized iPSCs cultured on MEFs were injected intramuscularly into the hind limb of SCID/beige mice (Harlan Laboratories, Madison, WI). Three mice were injected per cell line, each with one 6-well plate of cells. Matrigel (BD Biosciences) was added at 1/3 total volume to the cell suspension prior to injection. Tumors formed at 5 to 12 weeks and were processed for hematoxylin and eosin staining and histological analysis by the McArdle Laboratory for Cancer Research (University of WI-Madison). All animal work was conducted according to relevant national and international guidelines under the approval of the Cellular Dynamics International Animal Care and Use Committee.

### Cardiac Differentiation

Cardiogenesis was induced via a cell aggregate method. Briefly, TiPS cells grown on MEFs were harvested with collagenase IV (Invitrogren) and cells grown on Matrigel were dissociated into single cell suspension using Sodium Citrate. The cell suspension was allowed to form aggregates in ultra-low attachment flasks in the presence of recombinant human hepatocyte growth factor (HGF) and/or zbFGF. Additionally, ROCK inhibitor H1152 was added to Matrigel-sourced cell suspensions. Beating aggregates were dissociated and stained for cardiac troponin T (cTnT) (Abcam, Cambridge, MA, clone 1C11) on days 14 to 15.

### Neuronal Differentiation

The neural differentiation of TiPS cells was performed as previously described [29]. Briefly, TiPS grown on MEFs were partially dissociated with collagenase IV and cultured in suspension as aggregates in Stemline Neural Stem Cell Expansion Medium (Sigma-Aldrich) supplemented with B27 supplement (Invitrogen), bFGF (100 ng/ml) and epidermal growth factor (100 ng/ml, Chemicon, Billerica, MA). Cultures were passaged weekly using a McIlwain tissue chopper. To induce neural differentiation, spheres were grown in neural induction medium (DMEM/F12 plus N2 supplement, Invitrogen) for one week and then plated onto poly-ornithine/laminin (Sigma-Aldrich)-coated coverslips in the same neural induction medium supplemented with cAMP (1 uM, Sigma-Aldrich), ascorbic acid (200 ng/ml, Sigma-Aldrich), brain-derived neurotrophic factor and glial cell line-derived neurotrophic factor (both 10 ng/ml, R&D Systems) for a further 3 weeks. The expression of neuronal maker beta III-tubulin was analyzed by immunofluorescence staining as previously described [30].

## Supporting Information

Figure S1 DNA Fingerprinting. Short Tandem Repeat (STR) analysis shows TiPS cell lines are identical to parent activated T-cells for all 15 allelic polymorphisms detected across the 8 STR loci analyzed. Representative data from two TiPS lines (TiPS L-1 and TiPS L-2) are shown.
(3.65 MB TIF)

Figure S2 Alkaline Phosphatase (AP) Staining. TiPS lines TiPS L-1 and TiPS L-2 are AP positive. Images were acquired on an HP Scanjet G3110 computer scanner.
(2.87 MB TIF)

Figure S3 TiPS cell lines display normal karyotype. TiPS cell lines “TiPS L-1” and “TiPS L-2” were grown for 6 passages on MEFs, and lines “TiPS V-1” and “TiPS V-2” were grown on Matrigel for 8 of 18 total passages and 30 of 34 total passages, respectively. Cells were subjected to G banding analysis and no clonal abnormalities were detected.
(4.70 MB TIF)

Figure S4 Vector map of the MMLV retroviral construct used for reprogramming experiments. “RG” denotes reprogramming gene.
(1.91 MB TIF)
