# Supplementary figures and images for: Derivation of Induced Pluripotent Stem Cells from Human Peripheral Blood T Lymphocytes
Source: PLoS One. 2010 Jun 29;5(6):e11373. doi: 10.1371/journal.pone.0011373 (PMC2894062; doi:10.1371/journal.pone.0011373)

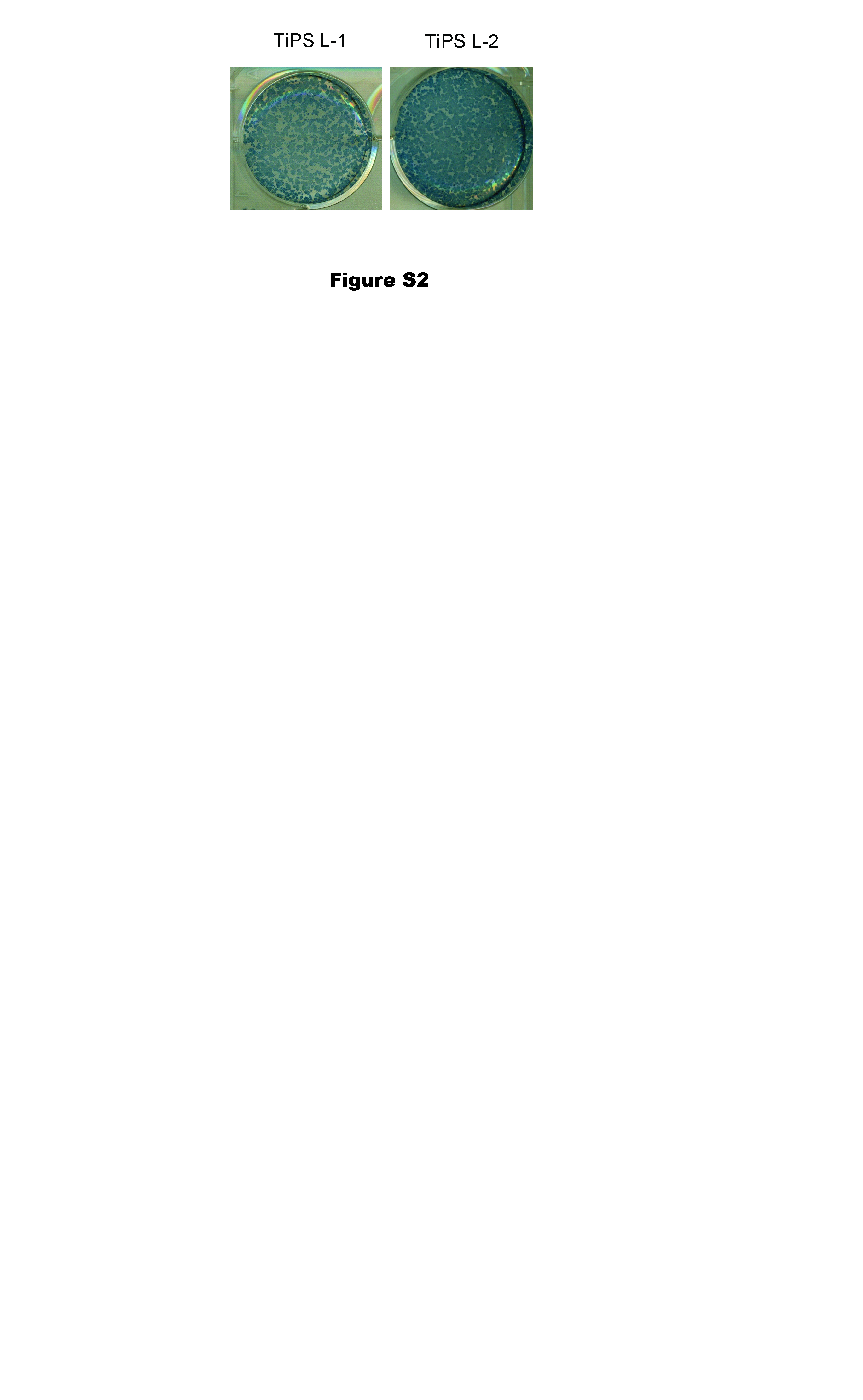

Supplement: Figure S2 — Alkaline Phosphatase (AP) Staining. TiPS lines TiPS L-1 and TiPS L-2 are AP positive. Images were acquired on an HP Scanjet G3110 computer scanner. (2.87 MB TIF) [file pone.0011373.s002.tif]
